# Evaluation of bonding of clear aligner attachments on aesthetic crown materials: a systematic review of *in vitro* studies

**DOI:** 10.3389/fdmed.2026.1888333

**Published:** 2026-07-28

**Authors:** Armaan Kaur, Crystal Runa Soans, M. S. Ravi, M. Shilpa

**Affiliations:** 1 Department of Orthodontics and Dentofacial Orthopaedics, Nitte (Deemed to be University), AB Shetty Memorial Institute of Dental Sciences (ABSMIDS), Mangalore, India; 2 Department of Public Health Dentistry, Nitte (Deemed to be University), AB Shetty Memorial Institute of Dental Sciences (ABSMIDS), Mangalore, India

**Keywords:** bond strength, bonding, clear aligner attachments, crowns, surface treatment

## Abstract

**Objectives:**

This study aims to assess the impact of various surface roughening treatments and bonding agents on aesthetic crown materials for the bonding of clear aligner attachments in orthodontic treatment. The primary objective is to assess their impact on shear bond strength, and the secondary objective is to assess their effect on surface roughness.

**Methods:**

This systematic review of literature (2015–2025) followed the Preferred Reporting Items for Systematic reviews and Meta-Analyses (PRISMA) guidelines. Data were collected from scientific articles investigating the impact of various surface roughening procedures on the bond strength and surface roughness for clear aligner attachments bonded to aesthetic crown materials. The articles were retrieved from three databases: Scopus, PubMed, and Web of Science. Risk of bias was assessed using the QUIN bias assessment tool for dental *in vitro* studies.

**Results:**

The results of the included articles were organised into tables in Word under the headings of author, country, material used, intervention(s), and results. The findings showed a positive increase in bond strength and surface roughness following surface treatments.

**Conclusion:**

This systematic review concludes that etching with hydrofluoric acid for 1 min and bonding with Filtek Z350 composite resin result in relatively higher bond strength and surface roughness. These findings may assist orthodontists in enhancing the bond strength of clear aligner attachments to aesthetic crown materials, hence improving orthodontic tooth movement. No external or internal funding was received for this review. It is registered with PROSPERO under the registration ID CRD420251174624.

**Systematic Review Registration:**

https://www.crd.york.ac.uk/PROSPERO/view/CRD420251174624, identifier CRD420251174624.

## Introduction

In 1945, Kesling (1) introduced the tooth positioner, a combination of an active treating appliance and an efficacious retainer. Since then, technology has advanced considerably, with Align technology unlocking its commercial advantages by utilising appropriate materials and three-dimensional computer-aided design to pre-plan the required tooth movements and fabricate clear aligners for patients (2). Patients have increasingly chosen clear aligner therapy (CAT) because of its aesthetic appeal (3). While the flexibility of aligner materials contributes immensely to patient comfort, it also limits the effectiveness of force application (4). Aligners have incorporated various auxiliaries, such as bite ramps, power ridges, and attachments, to increase their efficiency.

Attachments come in different shapes and sizes, are tooth-coloured, and are bonded to teeth, effectively increasing the grip of the aligners on the dentition (5). The increased grip is predominantly due to the increase in contact area, as well as effective force application being closer to the centre of resistance (6). Bodily movement of posterior teeth, rotational movements of premolars and canines, and extrusion of maxillary anterior teeth necessitate the use of attachments (7). Complex orthodontic movements, which limited the use of aligners previously, are now possible due to significant progress in auxiliaries (8).

Dentists use a variety of materials for the rehabilitation of missing teeth, implants, and endodontically treated teeth. Most commonly, monolithic zirconia and glass ceramics (silica-based) are used currently. Zirconia poses a significant challenge for bonding due to its chemical inertness and acid resistance (9, 10). The use of these materials hinders the predictability of tooth movement and poses a challenge for orthodontists. With the shift towards advanced digital workflows and 3D printing, emphasis needs to be placed on the bonding of attachments to crowns (11, 12).

This review aims to summarise and systematically organise the current limited literature on bonding clear aligner attachments to aesthetic crown materials, making this approach easier for orthodontists.

Review objectives:

- What is the effect of using various surface roughening procedures and bonding agents on the bonding of clear aligner attachments to aesthetic crown materials?
- Primary objective: To assess the effect on bond strength.
- Secondary objective: To assess the effect on surface roughness.

## Materials and methods

### Search strategy

The Cochrane Handbook for Systematic Reviews of Interventions (version 6.5, 2024) (13) was consulted in formulating this systematic review, and the review is reported in accordance with the Preferred Reporting Items for Systematic reviews and Meta-Analyses (PRISMA) 2020 expanded checklist (14). The PROSPERO registration ID is CRD420251174624.

The population, intervention, comparison, outcome (PICO) format was used to formulate the research question.

The PICO question was: “What is the effect of various surface roughening procedures and bonding agents on the bonding of clear aligner attachments to aesthetic crown materials?” (Table 1).

**Table 1:** PICO question.

PICO Component	Description
P (Population)	*In vitro* studies involving clear aligner attachments on different crown materials
I (Intervention)	Different protocols of bonding clear aligner attachments to different crown materials will be assessed
C (Comparison)	No treatment done
O (Outcome)	Effect on bond strength

The literature search was conducted using three databases: Scopus, PubMed, and Web of Science. The “Topic” field was used for Web of Science, “Article title,” “Abstract,” and “Keywords” for Scopus, and “All Fields” for PubMed. Table 2 represents the search strategy for each database. The search was limited to articles published between 2015 and 2025.

**Table 2:** Search string.

Database	Search string	Hits
Scopus	TITLE-ABS-KEY ((“clear aligner*” OR “clear aligner attachment*” OR “aligner attachment*” OR “orthodontic attachment*” OR “removable orthodontic appliance*”) AND (“bond strength” OR “shear bond strength” OR bonding OR adhesive* OR “surface treatment” OR “surface roughness”) AND (ceramic* OR crown* OR zirconia OR “lithium disilicate” OR “esthetic crown material*” OR “aesthetic crown material*” OR resin OR “3D printed resin”)) AND PUBYEAR > 201	104
Web Of Science	TS = ((“clear aligner*” OR “aligner attachment*” OR “clear aligner attachment*” OR “removable orthodontic appliance*”) AND (“bond strength” OR “shear bond strength” OR bonding OR adhesive* OR “surface treatment*” OR “surface roughness”) AND (ceramic* OR crown* OR zirconia OR “lithium disilicate” OR “esthetic crown material*” OR resin* OR “3D printed resin*”))	79
PubMed	((“Clear Aligners” [Mesh] OR “clear aligner*” OR “aligner attachment*” OR “clear aligner attachment*” OR “orthodontic attachment*” OR “removable orthodontic appliance*”) AND (“Dental Bonding” [Mesh] OR “bond strength” OR “shear bond strength” OR bonding OR adhesive* OR “surface treatment” OR “surface roughness”) AND (“Ceramics” [Mesh] OR ceramic* OR crown* OR zirconia OR “lithium disilicate” OR “esthetic crown material*” OR “aesthetic crown material*” OR resin OR “3D printed resin”))	77

### Eligibility criteria

In-depth inclusion and exclusion criteria were established to identify the most relevant articles. The included articles were published in English and within the last decade (2015–2025). The detailed eligibility criteria are presented in Table 3.

**Table 3:** Eligibility criteria included within the study.

PICO component	Inclusion criteria	Exclusion criteria
Population	- *In vitro* studies involving clear aligner attachments on different crown materials	- *In vivo* or *in vitro* studies involving clear aligner attachments on natural teeth
Intervention(s)	Bonding clear aligner attachments to crowns is challenging, thus requiring various surface roughening procedures and bonding agents to improve bond strength Different protocols of bonding clear aligner attachments to different crown materials will be assessed: - hydrofluoric acid - phosphoric acid - sandblasting - laser - carbide and diamond burs - various bonding agents	
Comparison(s) or control(s)	- No treatment done	- Studies involving natural teeth
Outcome(s)	The impact of various surface roughening procedures and bonding agents on the bonding of clear aligner attachments to different crown materials: - effect on bond strength - effect on surface roughness	Any studies that are excluded due to the selection criteria
Study design	- Available in the English language. - Full-text access through institutional account - Published within the last decade	- Available in languages other than English. - Full-text access not available - Published systematic reviews and meta-analyses - Studies including human and animal trials

### Data collection process

A multistage screening exercise, beginning with the title and abstract and followed by the full-text review, was carried out by the operator (AK). Due to differences in data interpretation and the application of inclusion and exclusion criteria, the search was performed by two independent authors (AK and CS). A third author (MR) was consulted in case of disagreements. Data were screened using Rayyan software, and referenced were managed using Zotero software.

### Results

#### Study selection

**Figure 1:**
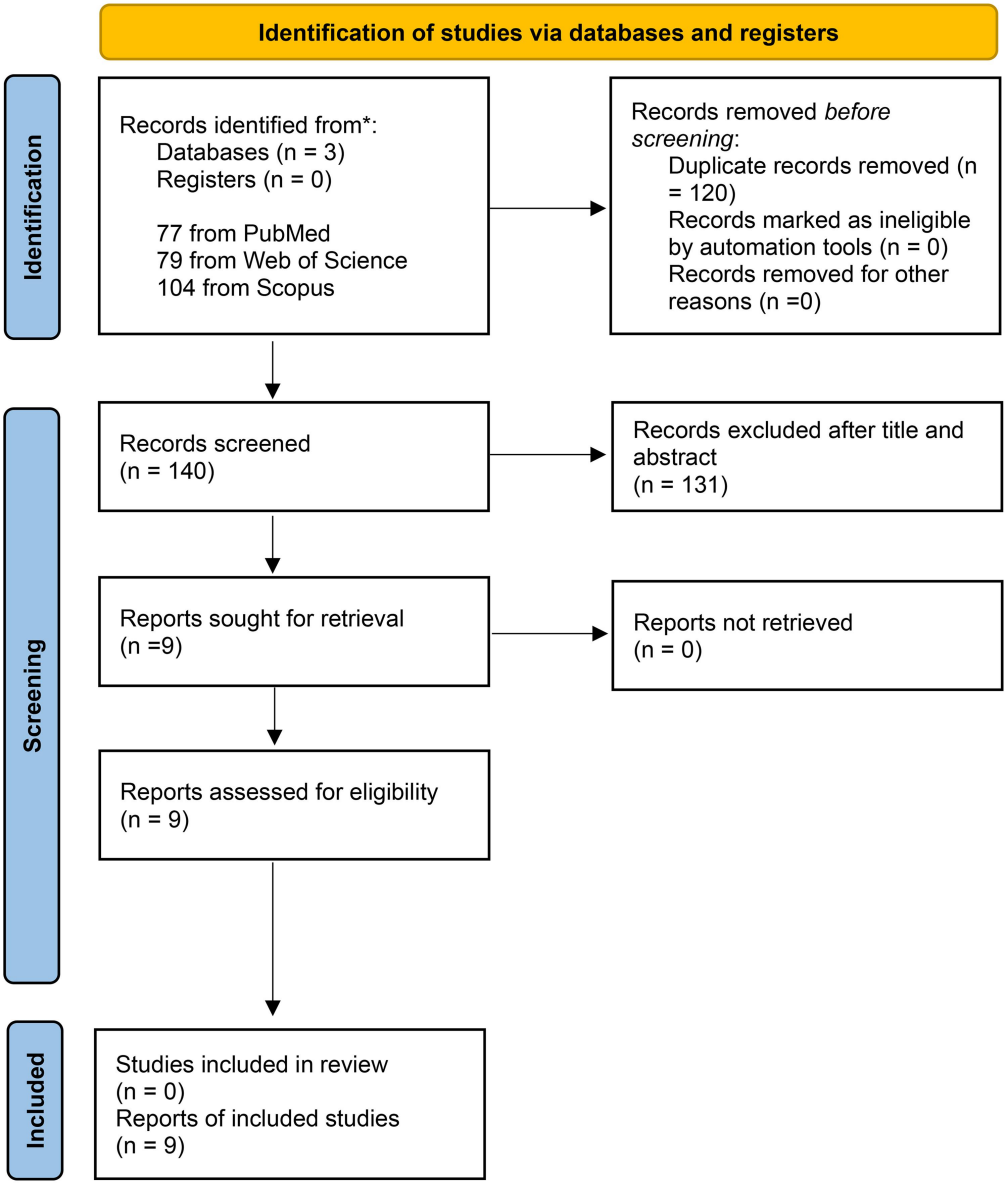
PRISMA flow diagram.

Initially, the search yielded 260 articles: 77 from PubMed, 79 from Web of Science, and 104 from Scopus. After removing duplicates, we were left with 140 articles. After title and abstract screening, we were left with nine articles to evaluate in the study. Figure 1 depicts the PRISMA 2020 flow diagram (14), illustrating the study selection.

#### Risk of bias within the studies

AK and CS performed the risk of bias assessment, and MR was consulted in cases of disagreement. The QUIN risk of bias assessment tool for *in vitro* dental studies was used to assess all nine articles and is illustrated in Table 4. According to the QUIN tool, the individual risk of bias was calculated using the following formula:

$$\begin{array}{rl} & \mathrm{Final}\;\mathrm{score}=\\ & \left(\mathrm{Total}\;\mathrm{score}\times 100)/(2\times \mathrm{Number}\;\mathrm{of}\;\mathrm{applicable}\;\mathrm{criteria}\right) \end{array}$$

and classified as low, medium, or high.

**Table 4:** QUIN assessment tool of individual studies.

Author	Clearly stated objectives	Detailed explanation of sample size	Detailed explanation of the sampling technique	Details of the comparison group	Detailed explanation of methodology	Operator details	Randomization	Method of measurement of outcome	Outcome assessor details	Blinding	Statistical analysis	Presentation of results	Final score (%)	Risk of bias
Alsaud et al. (15)	2	2	2	1	2	1	1	2	1	0	2	2	75	Low risk
Alhunayni et al. (17)	2	2	2	0	2	0	1	2	1	0	2	2	66.66	Medium risk
Criollo-Barrios et al. (22)	2	1	2	2	2	1	2	2	0	0	2	2	75	Low risk
Nitasnoraset et al. (18)	2	2	2	1	2	1	1	2	1	0	2	2	75	Low risk
Shahin et al. (23)	2	2	2	2	2	2	1	2	1	0	2	2	83.33	Low risk
Fayyad et al. (16)	2	2	2	1	2	0	2	2	1	0	2	2	75	Low risk
Nguyen et al. (19)	2	2	2	0	2	0	1	2	1	0	2	2	66	Medium risk
Çokakğlu et al. (21)	2	2	2	2	2	1	0	2	0	0	2	2	70.83	Low risk
Arslan et al. (20)	2	2	2	1	2	0	1	2	1	0	2	2	70.83	Low risk

## Results

The results are tabulated in six tables, with Table 5 summarising the primary effect on shear bond strength (SBS) across the various materials, and Table 6 elaborating on the surface roughness.

**Table 5:** Shear bond strength assessment.

Authors, year, country	Crown type, material	Intervention(s)			Sample size (*N*), groups	Results
		Surface roughening procedure	Bonding agent used	Composite used		
Alsaud et al., 2022, Saudi Arabia (15)	Lithium disilicate glass ceramics (IPS e.max crowns)	Etching with 9.6% HFA (*n* = 40) Etching with 37% PhA (*n* = 40) Air abrasion (AA) with Al_2_O_3_ (*n* = 40)	Single Bond Universal (SBU) (*n* = 20) Assure Universal Bond (*n* = 20)	3M Filtek Z350 (*n* = 10) Filtek Z350 Supreme Ultra (*n* = 10) flowable	*N* = 120 (*n* = 10/group) Group I: HFA + SBU + Filtek Z350 Group II: HFA + SBU + Filtek Z350 flowable Group III: HFA + AUB + Filtek Z350 Group IV: HFA + AUB + Filtek Z350 flowable Group V: PhA + SBU + Filtek Z350 Group VI: PhA + SBU + Filtek Z350 flowable Group VII: PhA + AUB + Filtek Z350 Group VIII: PhA + AUB + Filtek Z350 flowable Group IX: AA + SBU + Filtek Z350 Group X: AA + SBU + Filtek Z350 flowable Group XI: AA + AUB + Filtek Z350 Group XII: AA + AUB + Filtek Z350 flowable	**HFA (15.82** **±** **4.72 MPa)** **>** **AA (14.91** **±** **5.38 MPa)** under conditioning methods Least was with phosphoric acid **In bonding agents, AUB (14.0** **±** **6.04 MPa)** **>** **SBU (9.93** **±** **6.80 MPa)** **AA** **+** **AUB + Filtek Z350 gave the best results (21.80** **±** **3.86 MPa)** Lowest strength value was observed with PhA, followed by SBU with Filtek Z350 Supreme Ultra Flowable (0.96 ± 1.86 MPa)
Fayyadh et al, 2024, Egypt (16)	Lithium Disilicate	Sandblasting using 50 μm Al_2_O_3_ particles 9.5% HFA 37% PhA Er, Cr: YSGG laser		Filtek Z350 Filtek Z350 XT flowable	*N* = 80 (*n* = 10/group) Group I: Sandblasting using 50 μm Al_2_O_3_ particles for 5 s + Filtek Z350 Group II: Sandblasting using 50 μm Al_2_O_3_ particles + Filtek Z350 XT flowable Group III: 9.5% HFA + Filtek Z350 Group IV: 9.5% HFA + Filtek Z350 XT flowable Group V: 37% PhA + Filtek Z350 Group VI: 37% PhA + Filtek Z350 XT flowable Group VII: Er, Cr: YSGG + Filtek Z350 Group VIII: Er, Cr: YSGG + Filtek Z350 XT flowable	Within the different conditioning methods, **HFA (17.518 MPa)** > air abrasion > Er, Cr: YSGG > phosphoric acid (4.421 MPa) **Filtek Z350 (13.352 MPa)** > Filtek Z350 Supreme Ultra Flowable (11.306 MPa) **HFA + Filtek Z350 (18.60** **±** **2.05 MPa)** > HFA + Filtek Z350 Supreme Ultra Flowable (16.43 ± 2.09 MPa) > air abrasion + Filtek Z350 (15.00 ± 2.55 MPa) > PhA + Filtek Z350 Supreme Ultra Flowable (2.95 ± 1.16 MPa)
Alhunayni, 2025 Egypt (17)	Monolithic zirconia	9.5% HFA Air abrasion using 50-μm Al_2_O_3_ Er:Cr:YSGG treatment	Single Bond Universal adhesive was used for all groups	Fitlek Z250 Fitlek Z350	*N* = 66 (*n* = 11/group) Group I: HFA + SBU + Filtek Z250 Group II: HFA + SBU + Filtek Z350 Group III: sandblasted + SBU + Filtek Z250 Group IV: sandblasted + SBU + Filtek Z350 Group V: Er:CrYSGG laser + SBU + Filtek Z250 Group VI: Er:CrYSGG laser + SBU + Filtek Z350	In the **Filtek Z350 group, HFA (11.29** **±** **2.83)** > sandblast group (10.59 ± 2.76) > laser (8.33 ± 1.37) In Filtek Z250 group, **HFA group (10.57** **±** **2.49 MPa)** > sandblasting (10.31 ± 2.89 MPa) > laser treatment (8.29 ± 2.38 MPa)
Nitasnoraset, 2024, Thailand (18)	Monolithic zirconia	Surface treatment of all samples was first done using 9.6% HFA gel.	I: Si, using silane II: B, with bonding agent III: SiB, a combination of bonding agent and silane IV: SU, Superbond C&B resin cement V: SiSU, silane and Superbond C&B resin cement.	NA	*N* = 50 *n* = 10/group	Among all groups, SiSU showed the highest bond strength of 33.25 ± 4.57 MPa The least SBS value of (4.55 ± 0.99 MPa) was shown by the bonding agent group
Nguyen et al, 2025, Vietnam (19)	Full- contour zirconia crowns	Based on sand blasting duration: 10 s 30 s 60 s	According to the primer type: Silane MDP Universal	NA	*N* = 108 (*n* = 12/subgroup) Group I: sandblasting for 10 s + silane Group II: sandblasting for 10 s + MDP Group III: sandblasting for 10 s + Universal Group IV: sandblasting with 30 s + silane Group V: sandblasting with 30 s + MDP Group VI: sandblasting with 30 s + Universal Group VII: sandblasting with 60 s + silane Group VIII: sandblasting with 60 s + MDP Group IX: sandblasting with 60 s + Universal	The highest SBS was observed with **MDP at 30 s sandblasting (12.02** **±** **2.30 MPa)**, whereas **sandblasting was done for 60 s for the universal group to yield the greatest value (14.56** **±** **2.37 MPa)** The silane group with 10 s of sandblasting yielded the lowest SBS MDP and Universal primers constantly surpassed silane at all times
Arslan et al., 2023, Turkey (20)	Monolithic Zirconia	Fine cylindrical diamond bur Sandblasting with 50 µm Al_2_O_3_	Silane was used for all groups	Packable composite Filtek Z250 Flowable composite Filtek Supreme Ultra Flowable	*N* = 102(*n* = 17/subgroup) Group I: Filtek Z250 + silane + diamond bur Group II: Filtek Z250 + silane + AA with 50 µm Al_2_O_3_ Group III: Filtek Z250 + silane + AA with 110 µm Al_2_O_3_ Group IV: Filtek Supreme Ultra Flowable + silane + diamond bur Group V: Filtek Supreme Ultra Flowable + silane + AA with 50 µm Al_2_O_3_ Group VI: Filtek Supreme Ultra Flowable + silane + AA with 110 µm Al_2_O_3_	**Packable composite Filtek Z250** **+** **AA with 110 µm (3.32 MPa)** > Filtek Z250 + diamond bur (3.19 MPa) > Filtek Supreme Ultra Flowable + AA with 110 µm (2.22 MPa) The least value was observed for flowable composite and diamond bur (1.58 MPa)
Çokakoğlu et al, 2024, Turkey (21)	Lithium disilicate and Monolithic zirconia	Group I underwent etching, group II and IV were sandblasted.	Universal ceramic primer (Monobond Plus) Self-etching ceramic primer (monobond etch and prime MEP)		*N* = 40 (*n* = 10/group) Group I: LD + HFA + Monobond Plus (MP) Group II: MZ + AA + MP Group III: LD + MEP Group IV: MZ + AA + MEP	Treatment of lithium disilicate with HFA and MP yielded the least bond strength (6.02 ± 2.19 MPa) MZ + AA + MP gave the highest value (14.20 ± 6.26 MPa)
Criollo-Barrios, 2025, México (22)	3D printed definitive dental resin	No treatment (control), Etching with 4% HFA for 60 s AA with 50 µm Al_2_O_3_ particles	TXT—TransbondTM XT Light Cure Adhesive Primer SBU—SingleBondTM Universal adhesive	NA	*N* = 72 (*n* = 12/group) Group I: no treatment + TXT Group II: no treatment + SBU Group III: 4% HFA + TXT Group IV: etching with 4% HFA + SBU Group V: AA with 50 µm Al_2_O_3_ + TXT Group VI: AA with 50 µm Al_2_O_3_ + SBU	Al_2_O_3_ abrasion with SBU outperformed all (14.84 ± 2.24 MPa), and with TXT, observed a value of (10.36 ± 1.76 MPa) Hydrofluoric acid followed by higher SBS values than TXT Lowest SBS values were observed for the control group
Shahin et al., 2024, Saudi Arabia (23)	Bis-acryl composite material	No surface treatment Diamond bur Carbide bur Sandblasting with 50 μm Al_2_O_3_ Non-thermal plasma treatment (NTPT) Er:YAG laser		TXT (Transbond XT) was used for bonding	*N* = 120 *n* = 20/group	**Plasma (10.69** **±** **3.56 MPa)** > Er:YAG (9.68 ± 2.03 MPa) and sandblasting (9.15 ± 3.29 MPa), all increased SBS values Lowest SBS values were observed with the carbide and diamond burs

HFA, hydrofluoric acid; PhA, phosphoric acid; AA, air abrasion; SBU, Single Bond Universal; AUB, Assure Universal Bond; TXT, TRANSBONDT M XT; MPa, megapascal; Si, silane; SiSU, silane and Superbond C&B resin; NTPT, non-thermal plasma treatment; MZ, monolithic zirconia; LD, lithium disilicate; MDP, 10-methacryloyloxydecyl dihydrogen phosphate; SBS, shear bond strength. Bold values indicate the relatively higher bond strength values.

**Table 6:** Surface roughness assessment.

Authors	Country	Crown type, material	Intervention(s)	Sample size (N), groups	Results
Alsaud et al. (15)	Saudi Arabia	Lithium disilicate glass ceramics (IPS e.max CAD)	No treatment 9.6% HFA 37%PhA Air abrasion (AA) using 50 µm Al_2_O_3_	*N* = 60 *n* = 15/group	Least Ra exhibited by the no-treatment group (0.24 ± 0.08 µm) An increase was noted for PhA, and a further increase was observed with HFA Air abrasion yielded the highest value (1.20 ± 0.30 µm)
Fayyadh et al. (16)	Egypt	Lithium disilicate glass ceramic (IPS e.max Press)	Sandblasting with 50 μm Al_2_O_3_ 9.5% HFA. 37% PhA. Er, Cr: YSGG laser	*N* = 80 *n* = 20/group	The highest average surface roughness of 1.253 ± 0.12 μm was observed in the sandblasting group PhA had the least Ra of 0.399 ± 0.08 μm
Alhunayni et al. (17)	Egypt	Monolithic zirconia	9.5% hydrofluoric acid (HFA) Sandblasted with 50 μm Al_2_O_3_ Er:Cr:YSGG laser treatment	*N* = 66 *n* = 22/group	The highest surface roughness was seen in the HFA group (0.82 µm) > air abrasion (0.67 µm) > laser (0.63 µm)
Nguyen et al. (19)	Vietnam	Full- contour zirconia crowns	Based on sand blasting duration: 10 s 30 s 60 s	*N* = 144 (*n* = 48/group) Group I: Sandblasting for 10 s Group II: Sandblasting for 20 s Group III: Sandblasting for 30 s	The mean Ra for each group was 2.46 ± 0.40 μm (10 s) 3.28 ± 0.63 μm (30 s) 2.92 ± 0.53 μm (60 s) The highest surface roughness was obtained with sandblasting for 30 s
Criollo-Barrios et al. (22)	Mexico	3D printed definitive dental resin	No surface treatment 4% HFA airborne abrasion (AA) with 50 µm Al_2_O_3_	*N* = 36 *n* = 12/group	The highest mean surface roughness was demonstrated by the AA group (2.21 ± 0.30 µm) 4% HFA (Ra = 0.81 ± 0.20 µm) was lower The lowest roughness values were seen in the control group (Ra = 0.07 ± 0.03 µm)

HFA, hydrofluoric acid; PhA, phosphoric acid; AA, air abrasion; Ra, surface roughness.

### Shear bond strength assessment

Of the nine articles analysed in this review, all evaluated the effect of various surface roughening procedures on aesthetic crown materials. For lithium disilicate, Alsaud (15) reported that sandblasting, Assure Universal Bond (AUB), and Filtek Z350 resulted in the highest bond strength, followed by hydrofluoric acid (HFA), AUB, and Filtek Z350. Among the various surface treatments, etching with 9.6% hydrofluoric acid resulted in the highest SBS, followed by air abrasion. Etching with PhA exhibited the lowest bond strength. Fayyadh et al. (16) concluded that the combination of hydrofluoric acid and Filtek Z350 gave the best results, with hydrofluoric acid outperforming the other surface roughening procedures on lithium disilicate.

Alhunayni (17) reported that, for monolithic zirconia, the highest shear bond strength was achieved with HFA and Filtek Z350, followed by air abrasion with the same composite. In the Filtek Z250 group, etching with hydrofluoric acid also resulted in the highest bond strength. Etching with 9.5% hydrofluoric acid produced the highest mean SBS. Nitasnoraset (18) treated monolithic zirconia by etching with 9.6% hydrofluoric acid and, among primers, reported the highest bond strength with silane and Superbond in combination. Universal primer with 30 s of sandblasting produced the highest bond strength, followed by methacryloyloxydecyl dihydrogen phosphate (MDP) with 60 s of sandblasting on zirconia crowns (19). Packable composite Filtek Z350 and sandblasting with 110 µm Al_2_O_3_ produced better results than surface conditioning with a diamond bur (20). Another study conducted in Turkey reported that the highest bond strength was achieved by surface roughening monolithic zirconia using sandblasting and using Monobond Plus as the bonding agent (21).

On 3D-printed definitive resin, sandblasting with 50 µm Al_2_O_3_ reported higher SBS values than etching with HFA. Better bonding was also achieved with the Single Bond Universal adhesive than the Transbond Light-Cure Adhesive primer (22). Alumina blasting, plasma treatment, and Er:YAG laser treatment all successfully increased the bond strength (23).

### Surface roughness assessment

Surface roughness was evaluated in five of the nine studies included in this review and was the secondary outcome. Surface treatment procedures increased surface roughness; the maximum increase was seen with air abrasion using 50 µm Al_2_O_3_ on lithium disilicate, followed by hydrofluoric acid and phosphoric acid etching. Phosphoric acid etching yielded a very low surface roughness value, while the lowest surface roughness was observed in the control or no-treatment group (15, 16). In contrast, on monolithic zirconia, etching with hydrofluoric acid was more effective, followed by sandblasting and laser treatment (17). On 3D-printed definitive resin, the highest *R*_a_ was observed with air abrasion, followed by hydrofluoric acid. The lowest surface roughness was observed in the control group (22). On zirconia crowns, sandblasting for 30 s produced the best results, followed by 60 and 10 s (19).

## Discussion

With the increasing popularity of CAT, greater importance is needed to ensure its predictability and efficiency. Clear aligner attachments are one such area of interest in which treatment effectiveness can be improved, as they help control the forces acting on the teeth. Individual attachments are bonded to specific teeth based on their function in retention or as catalysts for tooth movement. Accordingly, they can either be classified as either passive (retentive) or active (24). These functions make it essential for orthodontists to achieve high bond strength when bonding clear aligner attachments.

With the advent of computer-aided design and manufacturing, milling machines have enabled an increase in zirconia and glass-ceramic crowns (25, 26). The ease of chairside milling makes these crowns the more convenient choice for both the patient and the dentist. The readily etchable surface of lithium disilicate (due to the presence of silica particles) makes bonding simpler than the stable nature of zirconia (27). Previously, studies have reported efficient results with air abrasion and the use of zirconia primer for zirconia crowns, while for lithium disilicate crowns, the composition of luting cement also holds importance (28–30).

Patients prefer aligner therapy chiefly due to aesthetics; this same aesthetics comes into play when patients choose tooth-coloured crowns for replacing missing teeth as well. Generally, bonding of attachments on crowns is discouraged unless it is necessary for treatment. Orthodontists inevitably need to determine how to achieve high bond strength when bonding clear aligner attachments to aesthetic crown materials, as one cannot predict the placement of attachments based on the presence of crowns.

According to both studies on lithium disilicate, the authors concluded that etching with hydrofluoric acid yielded the highest bond strength, while phosphoric acid consistently yielded the lowest. Surface roughness was highest following air abrasion. Filtek Z350 repeatedly outperformed Filtek Z350 Ultra Flowable (16). Assure Universal Bond demonstrated higher strength than Single Bond Universal on lithium disilicate (15).

On monolithic zirconia, hydrofluoric acid etching exhibited the highest bond strength and surface roughness, followed by sandblasting and laser treatment. Similar results were seen within the Filtek Z350 and Filtek Z250 groups, with Filtek Z350 surpassing Filtek Z250 (17).Sandblasting with 50 µm Al_2_O_3_ yielded better results than sandblasting for 60 s, and MDP was preferred over the universal and silane primer (19). Packable composite Filtek Z250 outperformed flowable composites (20). The combination of silane and Superbond C&B resin cement also showed promising results (18).

Criollo-Barrios et al. (22) conducted similar tests on 3D-printed definitive resin, in which Al_2_O_3_ sandblasting and Superbond Universal established their supremacy. Carbide and diamond burs did not increase bond strength significantly, while non-thermal treatments with plasma, an Er:YAG laser, and alumina sandblasting all increased bond strength in bis-acryl composite materials (23).

Innovation in this field has led to the development of a one-piece crown with prefabricated attachments. It is an interesting alternative when prosthodontic rehabilitation and orthodontic alignment are required. Current studies indicate that higher strength is achieved with 3D-printed crowns with attachments (31). In dental practice, depending upon the requirements of the patient, it is a viable option to provide a crown with an in-built attachment. It significantly improves the chances of optimum tooth movement and is an efficient multidisciplinary approach (32).

From a clinical standpoint, these findings are particularly relevant for both orthodontists and restorative dentists managing the increasing number of adult patients who present with, or require, aesthetic crown restorations during clear aligner therapy. As clear aligner attachments are routinely bonded directly onto crown surfaces to provide additional retention and more effective force application (33), the material composition and surface characteristics of the restoration can directly influence attachment stability and, consequently, the biomechanical predictability of tooth movement. This underscores the value of close interdisciplinary communication between orthodontists and restorative dentists when sequencing treatment, selecting crown materials, and planning the timing of attachment placement, particularly in adult patients, in whom comprehensive care often combines orthodontic and prosthodontic objectives (34, 35). For restorative dentists, awareness that hydrofluoric acid etching combined with a compatible composite resin, such as Filtek Z350, achieves the most reliable shear bond strength on lithium disilicate and zirconia substrates may guide material selection and surface preparation protocols when a crown is anticipated to receive an orthodontic attachment, while reinforcing the need for careful soft tissue isolation given the corrosive nature of hydrofluoric acid. For orthodontists, incorporating this evidence into clinical decision-making, ideally in consultation with the restorative team, may help reduce attachment failure rates, limit unnecessary chairside remakes, and support more predictable tooth movement in patients with aesthetic crown materials.

The findings of the present review suggest that increased surface roughness does not necessarily result in higher bond strength. While roughening procedures are traditionally employed to enhance adhesion through increased surface area and mechanical interlocking, several studies have demonstrated that the relationship between roughness and bond strength is not linear. Jennings was among the first to report that surface roughness alone could not adequately predict adhesive performance, emphasising the importance of the adhesive-substrate interaction rather than roughness in isolation (36).

Similarly, Uehara and Sakurai demonstrated that bond strength reaches an optimum at intermediate roughness levels, beyond which further increases in roughness may not improve adhesion and may even be detrimental. Excessive roughness can hinder complete adhesive penetration, create voids, and generate stress concentration sites that facilitate interfacial failure. These findings help explain why surface treatments producing the highest roughness values in the included studies did not consistently yield the highest bond strengths (37).

Another important consideration is that adhesion depends not only on surface topography but also on surface chemistry and wettability. Surface treatments, such as hydrofluoric acid etching and silanization, improve bonding by increasing surface energy and promoting chemical interactions between the restorative material and the adhesive. Budhe et al. (38) reported that bond strength is influenced by a combination of surface roughness, wettability, and adhesive characteristics, rather than roughness alone.

Furthermore, conventional roughness parameters such as average roughness (Ra) may not fully characterise the bonding surface. Likewise, Persson and Scaraggi proposed that excessive roughness may reduce the true microscopic contact area available for adhesion despite increasing the apparent surface area (39).

Collectively, these findings indicate that bond strength is a multifactorial phenomenon governed by an interplay of surface roughness, surface energy, wettability, chemical bonding, and adhesive properties. Therefore, the most effective surface treatment is not necessarily the one that produces the greatest roughness but rather the one that achieves an optimal balance between surface morphology and interfacial chemistry.

All studies included in this systematic review are *in vitro* studies and may yield variable results regarding the human oral microbiome. The use of clear aligners itself can alter the oral microbial environment and promote localised biofilm accumulation around tooth surfaces. Of clinical importance is the formation of biofilm at crown margins, where increased plaque retention may create a microenvironment conducive to bacterial colonisation, acid production, and adhesive interface degradation. This directly affects the durability of orthodontic attachments, as persistent biofilm accumulation at the attachment–tooth junction can compromise bond integrity through chemical and mechanical challenges, increasing the risk of marginal breakdown, debonding, and attachment loss over time. Consequently, meticulous marginal adaptation, effective oral hygiene, and regular monitoring are essential to minimise biofilm-related complications and support long-term bonding stability and attachment longevity during clear aligner therapy (40).

In addition to the oral environment, variations in laboratory aging procedures can significantly affect reported bond-strength values. Thermocycling, one of the most commonly employed artificial aging methods, subjects bonded specimens to repeated temperature fluctuations that simulate thermal stresses encountered intraorally. Repeated expansion and contraction of restorative materials, adhesives, and substrates can induce interfacial stresses, promote microleakage, and accelerate hydrolytic degradation, often resulting in reduced bond strength values (41–43). However, considerable heterogeneity exists among studies regarding thermocycling protocols, including the number of cycles, temperature range, dwell time, and transfer time, which may contribute to variability in outcomes and limit direct comparison between investigations.

Furthermore, differences in testing methodologies can substantially affect measured bond strengths. Variations in specimen preparation, bonding area, crosshead speed, storage conditions, loading configuration, and the use of shear vs. tensile bond-strength testing may yield different failure patterns and bond-strength values (44). Consequently, discrepancies in bond strength outcomes among studies may not necessarily reflect differences in the effectiveness of surface treatment protocols alone but may also arise from methodological heterogeneity. Therefore, aging procedures, thermocycling regimens, and testing methodologies should be carefully considered when interpreting and comparing bond strength results across studies.

A limitation of the present systematic review is the absence of studies evaluating the attachment bond strength of clear aligners to feldspathic porcelain, polymer-infiltrated ceramic networks (e.g., VITA Enamic), CAD/CAM PMMA, and other contemporary aesthetic crown materials. Consequently, conclusions regarding optimal surface-treatment protocols for these restorative materials could not be drawn. Future research should focus on investigating attachment bonding to these materials to expand the available evidence base and facilitate the development of material-specific clinical recommendations. It should also be noted that studies conducted by Alsaud et al. and Nitasnoraset et al. used 9.6% hydrofluoric acid, while Alhunayni et al. and Fayyadh et al. used 9.5% hydrofluoric acid. Across all four studies, the best results for shear bond strength were achieved with hydrofluoric acid etching (15–18). Only Criollo-Barrios evaluated 4% hydrofluoric acid, in which Al_2_O_3_ sandblasting preceded hydrofluoric acid etching (22).

This review is also subject to potential publication bias, as the literature search was confined to three electronic databases (Scopus, PubMed, and Web of Science) without a supplementary search of the grey literature, such as conference proceedings, theses, or unpublished data; this may have led to the exclusion of studies reporting null or unfavourable findings, potentially skewing the synthesised evidence towards positive treatment effects. In addition, eligibility was restricted to English-language publications, which may have excluded relevant studies published in other languages and introduced a further source of bias. Future systematic reviews in this field would benefit from broader database coverage, the inclusion of grey literature, and the removal of language restrictions to provide a more comprehensive and less biased synthesis of the available evidence.

Most studies had low risk of bias and were conducted thoroughly. Considerable clinical and methodological heterogeneity was observed among the included studies. Variations existed in restorative materials evaluated, surface conditioning methods, hydrofluoric acid concentrations, sandblasting protocols, bonding agents, composite materials used for attachment fabrication, sample sizes, and outcome assessment techniques. Furthermore, only five studies evaluated surface roughness in addition to shear bond strength. Due to these substantial differences in study design and interventions, quantitative meta-analysis was deemed inappropriate, and a narrative synthesis was performed instead as shown in Table 7.

**Table 7:** Sources of heterogeneity.

Source of heterogeneity	Variations observed across studies	Included studies	Impact on meta-analysis
Crown material tested	Lithium disilicate, monolithic zirconia, full-contour zirconia, 3D-printed definitive resin, bis-acryl composite provisional material	Alsaud et al., Fayyadh et al., Alhunayni et al., Nitasnoraset et al., Nguyen et al., Arslan et al., Çokakoğlu et al., Criollo-Barrios et al., Shahin et al.	Different substrates exhibit distinct chemical compositions, surface energies, and bonding characteristics, making direct comparison of pooled bond-strength values difficult
Surface treatment protocols	Hydrofluoric acid etching (4%–9.6%), phosphoric acid etching, air abrasion, laser treatment (Er:YAG, Er,Cr:YSGG), plasma treatment, diamond bur, carbide bur, no treatment	All studies	Interventions were fundamentally different and not evaluated uniformly across studies, preventing the generation of a common treatment effect
Hydrofluoric acid concentration and application	4%, 9.5%, and 9.6% hydrofluoric acid; varying application protocols	Alsaud et al., Fayyadh et al., Alhunayni et al., Criollo-Barrios et al., Nitasnoraset et al.	Different etching concentrations and protocols may produce significantly different surface characteristics and bond strengths
Air abrasion parameters	50 μm Al_2_O_3_, 110 μm Al₂O₃, varying durations and pressures	Alsaud et al., Fayyadh et al., Alhunayni et al., Arslan et al., Nguyen et al., Criollo-Barrios et al.	Differences in particle size and treatment duration influence surface roughness and bond strength outcomes
Bonding agents/primers used	Single Bond Universal, Assure Universal Bond, Monobond Plus, Monobond Etch & Prime, silane, MDP primer, Universal primer, Superbond C&B resin cement, Transbond XT primer	Most studies	Adhesive systems differ chemically and mechanically, creating incomparable intervention groups
Composite material used for attachments	Filtek Z350, Filtek Z350 XT Flowable, Filtek Z250, Filtek Supreme Ultra Flowable, various attachment composites	Alsaud et al., Fayyadh et al., Alhunayni et al., Arslan et al.	Different composite formulations influence attachment retention and bond strength independently of surface treatment
Outcome measurement groups	Studies compared different combinations of surface treatment, primer, and composite rather than a single standardised intervention	All studies	Absence of common comparator groups prevented calculation of a pooled effect size
Sample size distribution	Total sample sizes ranged from 40 to 144 specimens with subgroup sizes ranging from 10 to 48	All studies	Unequal subgroup allocation increases variability and reduces comparability across studies
Surface roughness assessment	Surface roughness assessed in only 5 of 9 studies, using different treatment protocols and reporting methods	Alsaud et al., Fayyadh et al., Alhunayni et al., Nguyen et al., Criollo-Barrios et al.	Insufficient uniformity and incomplete reporting precluded quantitative synthesis
Control groups	No-treatment controls present in some studies but absent in others	Criollo-Barrios et al., Shahin et al., Alsaud et al., others	Lack of a consistent control condition prevents standardised effect estimation
Testing conditions	Variations in specimen preparation, aging procedures, thermocycling protocols, and storage conditions	Across studies	Methodological differences introduce additional variability unrelated to the interventions themselves
Outcome reporting	Mean SBS values reported for different treatment combinations and different materials, often without a common reference group	All studies	Effect estimates could not be harmonised into a single meta-analytic model without introducing substantial bias

## Conclusion

Analysis of bond strength and surface roughness resulting from various surface treatments and bonding agents used for bonding helps an orthodontist decide which procedure to follow. Contrary to popular belief, higher surface roughness does not necessarily mean higher bond strength. Both lithium disilicate and monolithic zirconia studies concluded that surface conditioning with hydrofluoric acid and the use of Filtek Z350 yielded the best results, with sandblasting following closely. Sandblasting may not be the protocol of choice in clinical scenarios; our review highlights the efficacy of hydrofluoric acid, which compares favourably with sandblasting. In a clinical setting, the use of hydrofluoric acid is limited due to its highly corrosive nature. The importance of using hydrofluoric acid with caution or with a gingival barrier, such as a rubber dam, needs to be emphasised.

These conclusions, while clinically meaningful, must be interpreted with caution. This review includes a limited number of eligible studies, and considerable heterogeneity was present across the included studies with respect to the crown materials evaluated, surface roughening protocols, hydrofluoric acid concentrations, bonding agents, attachment composite materials, sample sizes, and outcome assessment methods; this heterogeneity precluded a quantitative meta-analysis. Furthermore, all of the evidence synthesised in this review originates from *in vitro* studies, which, despite standardised laboratory conditions, cannot fully reproduce the complex intraoral environment, including salivary contamination, cyclic thermal and mechanical loading during function, and interactions with the oral microbiome. Clinical extrapolation of these *in vitro* findings should therefore be undertaken, and further confirmation through well-designed *in vivo* and clinical studies is necessary before definitive clinical protocols can be established. While recognising the inherent limitations of the included studies, the most practical and clinically relevant recommendation has been derived from the best available evidence. These recommendations are intended to inform practice and highlight areas where further high-quality research is needed.

The unconventional approach of using a 3D-printed crown with an attachment remains a design that can be recommended for clinical use by orthodontists and prosthodontists, pending further *in vivo* validation.

## Data Availability

The original contributions presented in the study are included in the article/Supplementary Material, further inquiries can be directed to the corresponding author.
